# Supplementary material for: Differences in Placental Imprinted Gene Expression across Preeclamptic and Non-Preeclamptic Pregnancies
Source: Genes (Basel). 2020 Sep 29;11(10):1146. doi: 10.3390/genes11101146 (PMC7601230; doi:10.3390/genes11101146)
Supplement: Supplementary file 1 [file genes-11-01146-s001.pdf]

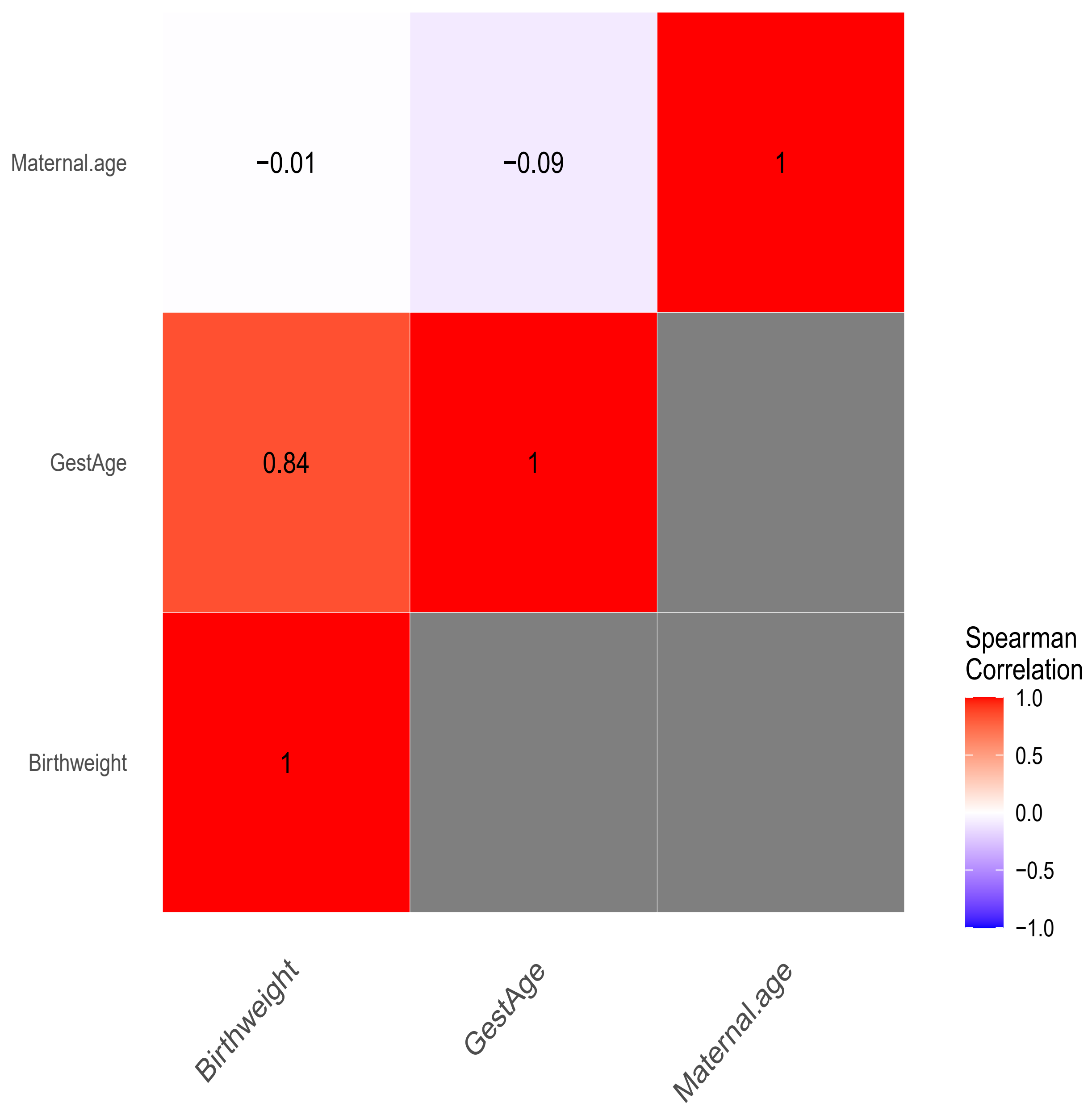

Figure S1. Correlation among continuous demographic variables. Gestational age and birthweight are significantly, positively correlated with one another ($p<0.01$)

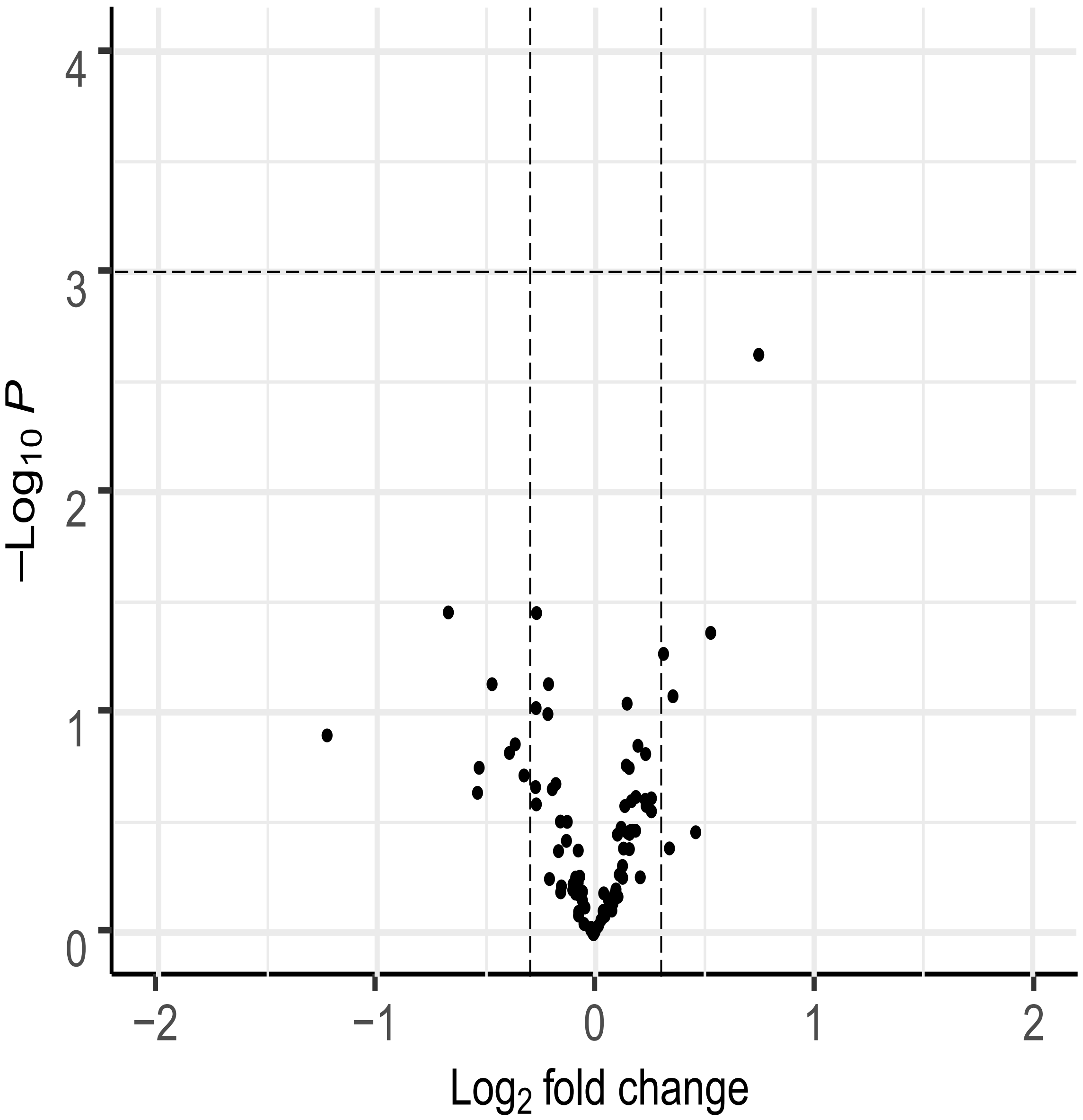

Figure S2. Differential gene expression analysis comparing preterm and term controls. Volcano plot depicting Log2 fold change values on the x-axis and -Log10 p-values on the y-axis. Points falling above the dashed horizontal line indicate genes that are significantly differentially expressed based on an FDR < 0.05. No differences in gene expression levels are observed comparing normotensive placenta delivered preterm and at term.

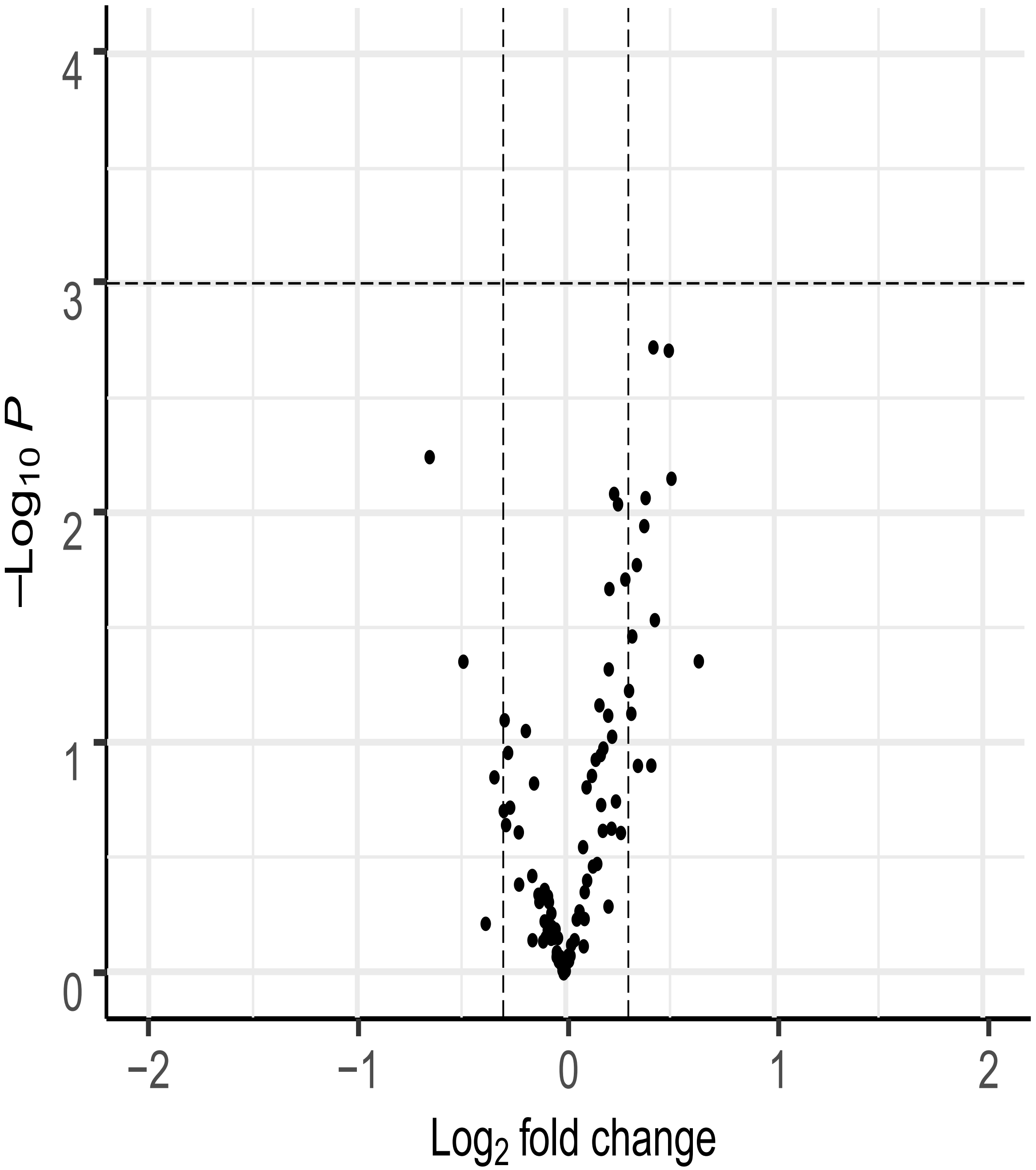

Figure S3. Differential imprinted gene expression analysis comparing male and female placentas from normotensive pregnancies delivered at term. Volcano plot depicting log2 fold change values on the x-axis and -log10 p-values on the y-axis. Points falling above the horizontal dashed line indicate genes significantly differentially expressed based on an FDR < 0.05. No significant sex differences in gene expression levels are observed.

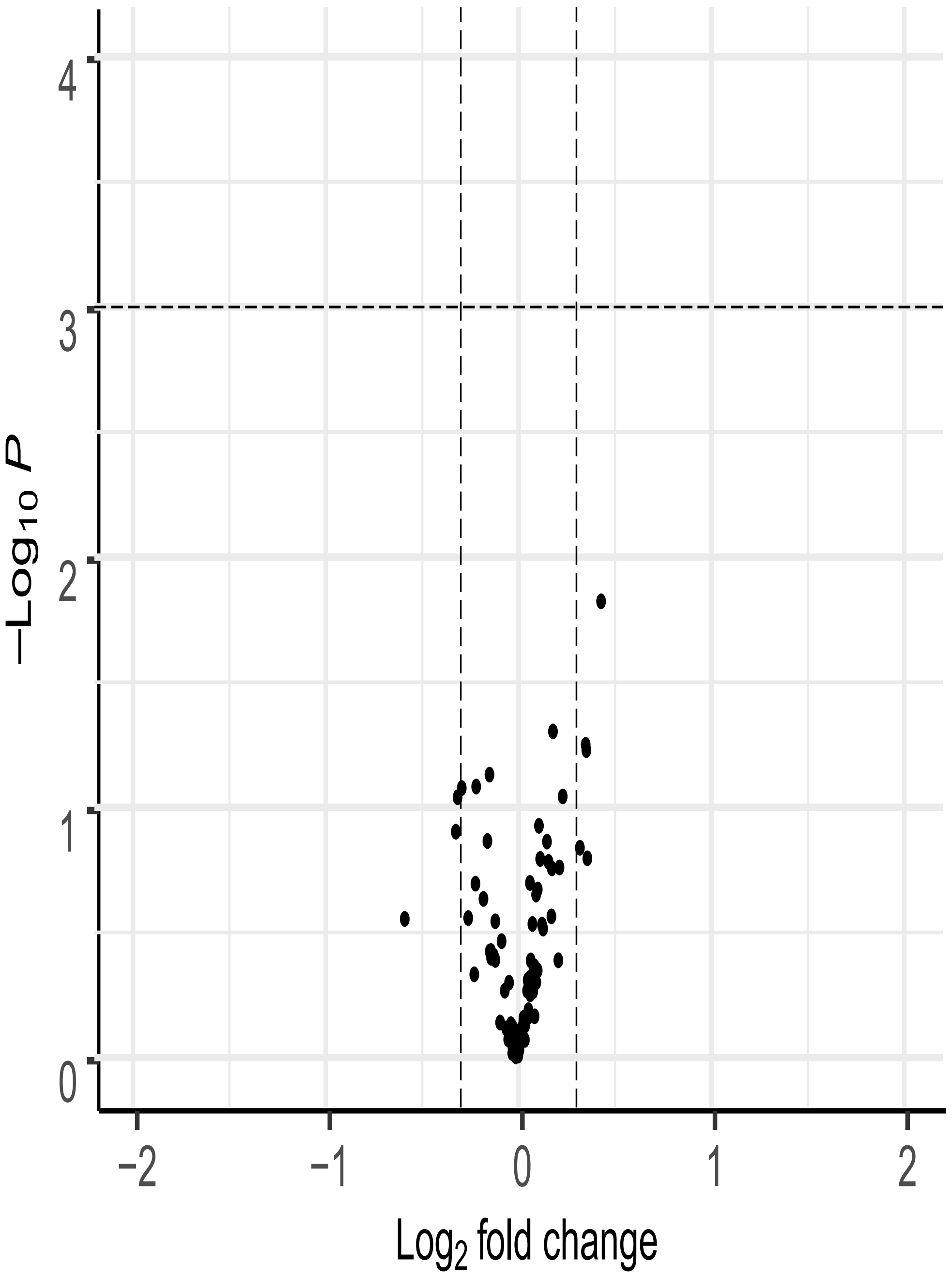

Figure S4. Differential placental imprinted gene expression analysis between late onset cases and term controls, restricted to those who underwent labor. Points falling above the horizontal dashed line indicate genes significantly differentially expressed based on an FDR < 0.05. No genes are differentially expressed between cases and controls.

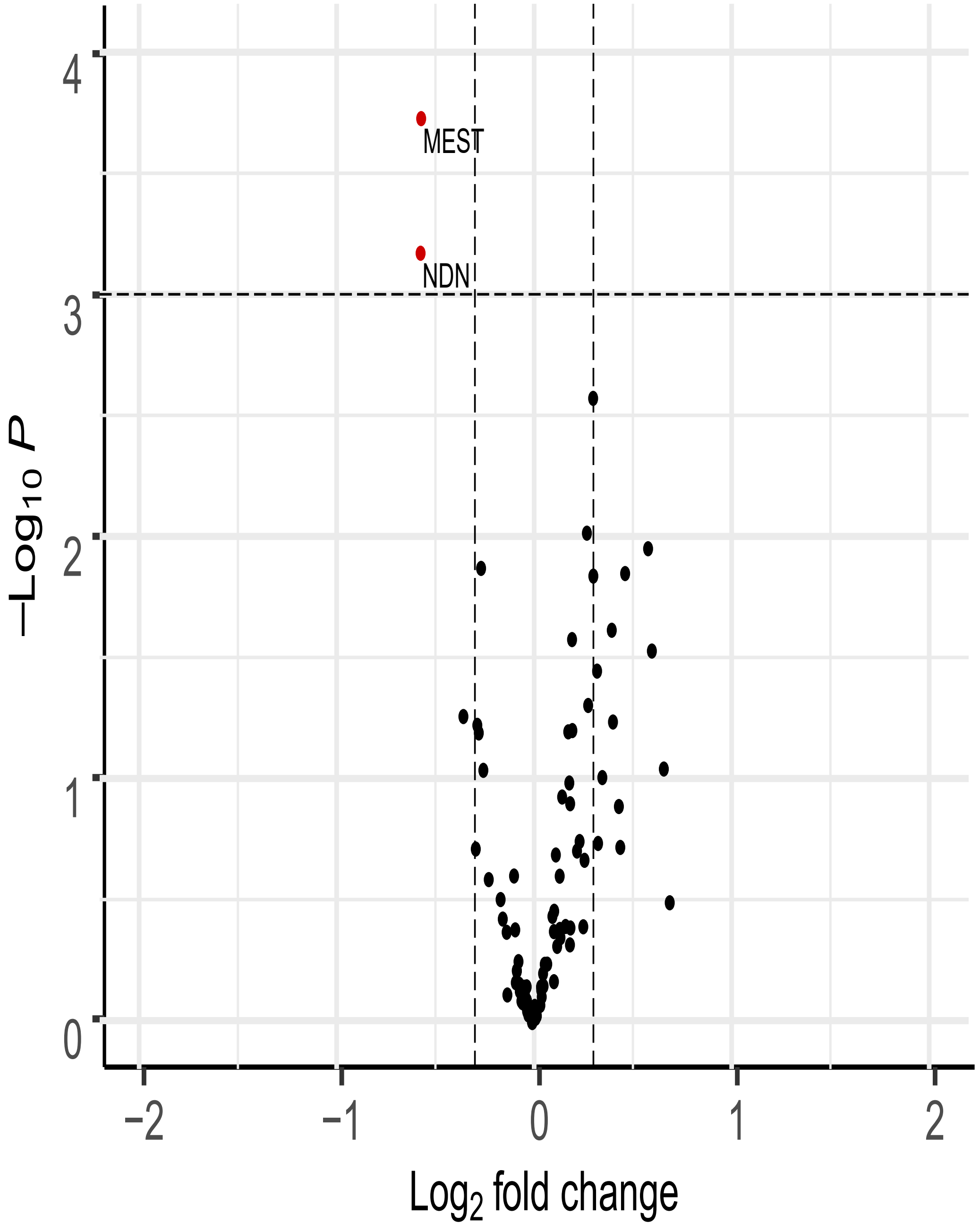

Figure S5. Differential placental imprinted gene expression analysis between early onset cases and preterm controls, restricted to those who underwent labor. The volcano plot depicts $\log_2$ fold change values between cases and controls on the x-axis and $-\log_{10}$ p-values on the y-axis. Points (in red) falling above the horizontal dashed line indicate genes that are significantly differentially expressed based on an FDR < 0.05. Two genes, MEST and NDN, are differentially expressed between cases and controls.

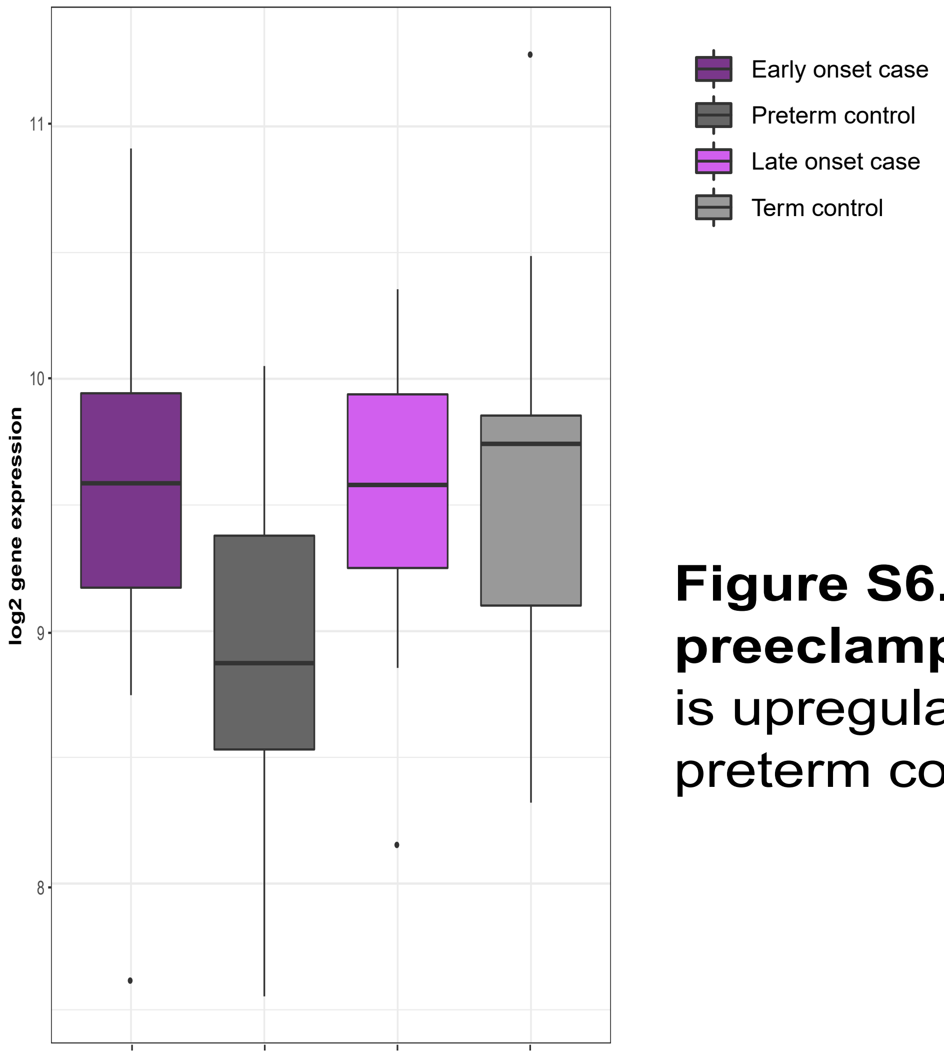

Figure S6. *DLX5* gene expression levels across preeclampsia cases and controls. *DLX5* gene expression is upregulated among early onset cases compared to preterm controls (FDR=0.15).
